# Supplementary material for: Patterns of Intron Gain and Loss in Fungi
Source: PLoS Biol. 2004 Nov 30;2(12):e422. doi: 10.1371/journal.pbio.0020422 (PMC532390; doi:10.1371/journal.pbio.0020422)
Supplement: Table S1 — Also available at http://genes.mit.edu/NielsenEtAl/. (4.3 MB ZIP). [file pbio.0020422.st001.zip › NielsenEtAl/html/448.html]

AN8187.1.NCU06679.1.MG07323.1.FG06798.1


```
 CLUSTAL W (1.82) Multiple Sequence Alignments - Introns Inserted


Sequence 1: NCU06679.1	564 aa
Sequence 2: MG07323.1	436 aa
Sequence 3: FG06798.1	423 aa
Sequence 4: AN8187.1	496 aa
Alignment Length: 650 aa
Number Identitical Residues: 212 aa
Alignment Score (without introns) 10946


MG07323.1 	------------------------------------------------------------
NCU06679.1	------------------------------------------------------------
FG06798.1 	------------------------------------------------------------
AN8187.1  	MSIKQKTCKRSLTGLAIHQDLNAIEPCWAWMKKRTTSRGAPRDKKTGEAEWRQAWADLPQ
          	 : ...:.. : :. :  .. .: ... :  .. ::: .:. ...:..:.  .: :. ..

MG07323.1 	------------------------MAPPPA~MD~V~DE1M~DVEED-FDQEQRIINEE1Y
NCU06679.1	------------------------MARDEI~VD~D~VD~V1NMEEDDAEAEQRLINEE~Y
FG06798.1 	------------------------------~MD~V1DM~I~HDEED--DQGERLINEE1Y
AN8187.1  	ETIQHWIERLIRHIQIVIELEGGNEYKEGL1IC1F~IE~E~QEEQEEERTEEKIINEE1Y
          	.: .   .      .   . ....       :         . *::.    :::**** *

MG07323.1 	KTWKKNSPFL~YDMILS2TALAWPTLTVQWLPDVKE~PEGKNYRIHRVLLGTHTSGATDE
NCU06679.1	KIWKKNSPFL~YDMMLS~TALEWPTLTTQWFPDVKN~PKDKSHTVHRLLLGTHTAEGKPN
FG06798.1 	KTWKKNSPFL2------~TALTWPTLTVQWFPDVKE~PEGKNYKIHRLLLGTHTSDESPN
AN8187.1  	KTWKKNAPFL~YDMILS~TALEWPTLTTQWLPDKQD2ASDKPYSTHRLLLGTHTSSDAQN
          	* ****:***  .   : *** *****.**:** :: ...* :  **:******:    :

MG07323.1 	YLQIAEVEIPKAVQPNPADYDEDRGEIGGYGGSKGS-EAAAIKWNITQKIDHPGEVNKAR
NCU06679.1	YLQIAEVEIPKMVELNPRDYDEERGEIGGYGSKASSGEPLCIRFKITQKIDHPGEVNKAR
FG06798.1 	YLQIADVQIPKVVAPNPDDYDEERGEIGGYGK---SGDVAAIKCDIVQRIEHPGEVNKAR
AN8187.1  	YLQIAQVQLPNPNAPNPDDYDEERGEIGGYGGS-SKKAPMEINFKIVQKIDHKGEVNKAR
          	*****:*::*:    ** ****:******** . ..     *. .*.*:*:* *******

MG07323.1 	~YQPQNPDIIATACVDGKILIFDRTKHSLQPS--GTPNPQYELVGHKAEGYGLNWSPHDE
NCU06679.1	~YQPQNPDIIATLAVDGRVLIFDRTKHSITPS--GTPSPQLELIGHKEEGFGLNWNPHEE
FG06798.1 	~YQPQNPDILATLCVDGKILIFDRTKHPLDPTSTGKVNAQIELVGHEAEGFGLNWNPHEE
AN8187.1  	2-----------------VMIWDRSKHQSVPT--GTVNPQMELLGHKQEGFGLSWSPHVA
          	                  ::*:**:**   *:  *. ..* **:**: **:**.*.**  

MG07323.1 	GCLVTG~SSDQTVLLW~DLKDVQPNNRILKPKRKYTHHSQVVNDVQYHPLVKHFIGTVSD
NCU06679.1	GCLVTG~SEDKTVLLW~DLKTYEGTSKQLKYSRKYTHHSHIVNDVQHHPLVKSWIGTVSD
FG06798.1 	GCLASG~SEDTTMRLW2DLKTLKADSRILNPSRTYRHHTQIVNDVQYHPISKNFIGSVSD
AN8187.1  	GHLATG1--------R~DLTTYTKNNKALQPVRTYTHHSSIVNDVQHHPLHSSLIGTVSD
          	* *.:*           **.     .: *:  *.* **: :*****:**: .  **:***

MG07323.1 	DLTLQILDTRQESNDKAALVAKNGHSDAINALDFCPASEFLVATASADKTIGLWDLRNVK
NCU06679.1	DLTLQIIDVRRPETDKAAIVARNGHSDAINALAFNPRVETIIATASADKTIGIWDMRNMK
FG06798.1 	DQTLQIVDIRHSETNKAAVVAKRGHLDAINALAFNPNSEVLVATASADKTIGIWDLRNVK
AN8187.1  	DITLQILDTRVAETTRAAATAEGQHRDAINAIAFNPAAETVLATGSADKTIGLWDLRNLK
          	* ****:* *  .. :** .*.  * *****: * *  * ::**.*******:**:**:*

MG07323.1 	DKIHTLEGHRDAVTSVFWHPHEAGILASGSYDRRILFWDLSRVGEEQQPDDAEDGPPEL2
NCU06679.1	SKVHTLEGHQDAVTSLEWHPTESAILGSGSYDRRLLFWDISRVGDEQTQDDAEDGPPEL~
FG06798.1 	EKVHTLEGHNDAVTSLAWHPTEAGILGSASYDRRIIFWDLSRVGEEQLPDDQDDGPPEL2
AN8187.1  	TKLHTLENHTDSVTSISWHPFEEAVLASASYDRKIMFWDLSRAGEEQTPEDAQDGPPEL2
          	 *:****.* *:***: *** * .:*.*.****:::***:**.*:**  :* :****** 

MG07323.1 	LFMHGGHTNHLADFSWNPNEPWMVCSAAEDNLLQVWKVAESIVKRDDADLPVDELGP---
NCU06679.1	LFMHGGHTNHLADFSWNRNDPWLVCSAAEDNLLQIWKVANSIVSKEPADMSTPELDDPKP
FG06798.1 	LFMHGGHTNHLADFSWNPNEPWLVASAAEDNLLQIWKVAESIVGKDDGDLPIDELDR---
AN8187.1  	LFMHGGHTNRISDFSWNLNDPWVLCSAAEDNLLQVWKVADAIVGKDMEDVPTEELEP---
          	*********:::***** *:**::.*********:****::** ::  *:.  **     

MG07323.1 	----~-------------------------------------------------------
NCU06679.1	KQSK1IQQQQEEQQQAHEKSAEQAATIGSRTEKGKGKEAPKEGSRLQDGTSGRDNGDGDD
FG06798.1 	----~-------------------------------------------------------
AN8187.1  	----~-------------------------------------------------------
          	                                                            

MG07323.1 	------------------------------------------------------------
NCU06679.1	DSEDTSASEQKGRGKSKGMSVYESYLRTVEEARRAAQMTGAERHRKTRSRTTFGAESQQK
FG06798.1 	------------------------------------------------------------
AN8187.1  	------------------------------------------------------------
          	                                                            

MG07323.1 	----
NCU06679.1	QQQQ
FG06798.1 	----
AN8187.1  	----
          	
```
